# Impact of home-based port infusion versus hospital-based peripheral infusion on anxiety, quality of life, and emergency visits in gastrointestinal cancer: a prospective observational study by the Turkish Oncology Group (TOG)

**DOI:** 10.1007/s00520-026-10790-z

**Published:** 2026-05-22

**Authors:** Ozan Ahmet Cetin, Turan Karaoglu, Ali Alkan, Tugba Yavuzsen, Ozgur Tanriverdi

**Affiliations:** 1 Department of Medical Oncology, Mugla Sitki Kocman University Faculty of Medicine, Mugla Universitesi Egitim Ve Arastirma Hastanesi, Onkoloji Poliklinigi, 48000 Mugla, Turkey; 2 Department of Medical Oncology, Dokuz Eylul University Faculty of Medicine, Izmir, Turkey

**Keywords:** Chemotherapy, adjuvant, Infusions, intravenous, Home care services, Ports and catheters, Anxiety, Quality of life, Gastrointestinal neoplasms

## Abstract

Home-based chemotherapy via port catheters offers logistical and potential psychological benefits but may pose unique challenges for patients undergoing prolonged regimens such as mFOLFOX6. Evidence on the association between home-based infusion and anxiety, quality of life, and healthcare use in gastrointestinal (GI) cancer patients remains limited. In this prospective study, 190 GI cancer patients scheduled for adjuvant mFOLFOX6 were enrolled between July 2020 and December 2024. Patients chose either home-based port infusion (*n* = 94) or hospital-based peripheral infusion (*n* = 96). Anxiety was assessed using the State-Trait Anxiety Inventory (STAI-1 and STAI-2), and quality of life via EORTC QLQ-C30 at baseline, after six cycles (~3 months), and after 12 cycles (~6 months). Treatment-related toxicities and emergency visits were recorded. Logistic regression identified factors associated with high anxiety, low quality of life, and emergency visits. Baseline characteristics were similar between groups except for younger age in the home-based group, which lost significance after Bonferroni correction. After six cycles, home-based patients showed significantly higher state and trait anxiety than hospital-based patients (*p* < 0.001), though differences diminished by 12 cycles. Home-based patients also reported worse dyspnea, pain, fatigue, and nausea/vomiting after six cycles, with most symptoms improving later. Emergency visits without organic pathology were more frequent in the home-based group (32% vs. 13%, *p* = 0.002), although this difference did not remain statistically significant after Bonferroni correction. Multivariable analysis identified home-based infusion, living alone, social inactivity, shorter treatment duration, and low quality of life as independent factors associated with high state anxiety. Similar factors were associated with high trait anxiety, low quality of life, and emergency visits. Home-based port infusion for mFOLFOX6 is associated with transient increases in anxiety and impairments in quality of life early in treatment, which diminished by the end of treatment. Psychosocial support and close follow-up may help reduce anxiety and unnecessary emergency visits, particularly for socially isolated patients. Further randomized studies are warranted.

**Supplementary Information:**

The online version contains supplementary material available at 10.1007/s00520-026-10790-z.

## Introduction

In recent years, home-based chemotherapy has gained increasing prominence in oncology owing to its potential to improve patient comfort, reduce hospital resource utilization, and lower overall healthcare costs [1]. Advances in central venous port catheter systems have enabled the safe administration of continuous infusion regimens in the home setting, offering patients greater flexibility and autonomy during prolonged treatment courses [2].

Among systemic treatment options for gastrointestinal (GI) malignancies, the modified FOLFOX6 (mFOLFOX6) regimen, comprising oxaliplatin, 5-fluorouracil (5-FU), and leucovorin, remains a cornerstone therapy in neoadjuvant, adjuvant, and metastatic settings, particularly for gastrointestinal tract cancers [3–7]. Nevertheless, the mFOLFOX6 regimen requires a 46-h continuous infusion, which introduces logistical challenges that may affect both patients and healthcare systems [8, 9]. Consequently, as in many healthcare systems worldwide, the use of implantable venous port systems has become increasingly common, despite variations in clinical practice related to institutional resources, technical infrastructure, and patient-related factors. In Türkiye, implantable port catheters are commonly used in patients requiring long-term intravenous chemotherapy, particularly in those receiving multi-cycle regimens such as mFOLFOX, although nationwide prevalence data are limited [10].

In clinical practice, chemotherapy may be administered via peripheral venous access or implantable port systems, each with distinct advantages and limitations. Peripheral access is generally suitable for short-term treatment but is associated with higher risks of phlebitis, extravasation, and repeated venipuncture. In contrast, implantable ports provide reliable long-term vascular access and facilitate the safe administration of vesicant agents and prolonged infusions. However, port implantation is an invasive procedure and carries risks such as infection, thrombosis, and mechanical complications, underscoring the need for appropriate patient selection and standardized management protocols [11–15].

Several studies have demonstrated the feasibility and safety of home-based chemotherapy delivered via implantable ports, reporting reductions in hospital stays, healthcare costs, and comparable treatment efficacy relative to inpatient administration [10–14]. Nonetheless, evidence regarding the psychological impact, adherence to treatment, and complication profiles of home-based chemotherapy, particularly for prolonged infusion regimens such as mFOLFOX6, remains limited and inconsistent [1, 14, 15].

Anxiety and health-related quality of life (HRQoL) are increasingly recognized as key patient-centered outcomes in oncology. Reported rates of clinically significant anxiety among patients receiving chemotherapy range from 30 to 50%, influenced by disease characteristics, treatment setting, and individual coping capacity [15]. Home-based chemotherapy may alleviate anxiety by reducing hospital visits and promoting a sense of normalcy, yet it may also heighten psychological distress due to reduced medical supervision, uncertainty in symptom interpretation, and responsibility for self-management. These factors may contribute to increased emergency department utilization, particularly when patients are uncertain about the severity of treatment-related symptoms or device-related complications [15–17].

Home-based chemotherapy in gastrointestinal cancers generally refers to the administration of prolonged intravenous infusion therapies outside the hospital setting, most commonly facilitated by implantable venous port catheter systems and ambulatory infusion pumps. This approach allows patients to receive continuous chemotherapy infusions at home after initial hospital-based administration, thereby reducing inpatient stay while maintaining treatment continuity. Accordingly, the prolonged nature of the mFOLFOX6 regimen further accentuates these challenges. Managing a 46-h continuous infusion, whether at home or in the hospital, can interfere with daily activities, disrupt sleep, and impose logistical burdens related to device handling, monitoring, and troubleshooting [18–20]. In the home setting, limited access to immediate professional support may intensify patient anxiety, whereas in-hospital administration may impose psychological and practical burdens associated with prolonged hospitalization. Collectively, these considerations highlight the importance of structured education, accessible clinical support, and individualized care strategies to optimize patient experience and safety [20].

Therefore, this study aims to compare home-based port infusion therapy with hospital-based infusion in patients receiving adjuvant mFOLFOX6 for gastrointestinal cancers, focusing on differences in anxiety levels, health-related quality of life, treatment-related toxicities, and the frequency of emergency department visits.

## Methods

### Study design and participants

This prospective observational study was conducted between 1 July 2020 and 30 June 2025. Patient recruitment was completed on 31 December 2024. All enrolled patients were subsequently followed for completion of their planned treatment courses and outcome assessments, including longitudinal evaluation of clinical parameters and patient-reported outcomes. The follow-up period extended until 30 June 2025 to allow sufficient time for completion of therapy and collection of post-treatment data, including toxicity profiles, anxiety measures, quality-of-life assessments, and healthcare utilization. The study protocol was approved by the Ethics Committee of Mugla Sitki Kocman University Clinical Research Ethics Commitee (Approval No. 200096/084, date: April 16, 2020) and conducted in accordance with the Declaration of Helsinki.

Eligible participants were adult patients aged 18 years or older who had been diagnosed with gastrointestinal (GI) cancers, undergone surgical treatment, and were scheduled to receive adjuvant mFOLFOX6 chemotherapy. Eligibility assessment and study enrollment were performed after completion of surgical treatment and prior to initiation of the first cycle of adjuvant chemotherapy, ensuring that baseline evaluations reflected pre-treatment status. Inclusion criteria required sufficient cognitive function and the absence of any impairments in language comprehension, perception, or speech. Exclusion criteria comprised patients who discontinued chemotherapy due to grade 3–4 toxicity, developed significant cardiopulmonary or cerebrovascular comorbidities preventing treatment continuation, or experienced complications related to the port catheter necessitating revision or removal, including catheter malfunction, thrombosis, infection, or abscess formation. All patients received comprehensive verbal and visual information regarding the study objectives and procedures, and written informed consent was obtained before enrollment.

The decision regarding port catheter placement was entirely patient-driven following thorough education about the potential advantages and risks of port use. Patients who declined port catheter placement or for whom placement was not feasible due to logistical challenges, such as scheduling delays or time constraints, were assigned to the hospital-based infusion group. Conversely, patients who underwent successful port catheter insertion were treated via home-based infusion therapy. Randomization was not feasible because the decision regarding port catheter placement involved patient preferences and ethical considerations, given the invasive nature of the procedure. Accordingly, the study was conducted as a non-randomized prospective observational design, and efforts were made to keep the comparison groups as similar as possible in baseline characteristics. This patient-driven allocation approach may introduce selection bias, as treatment groups were not randomly assigned.

The primary aim of the study was to compare anxiety levels, quality of life, treatment-related toxicities, and the frequency of emergency department visits between patients receiving home-based versus hospital-based infusion of adjuvant mFOLFOX6 therapy.

Data were collected prospectively using a combination of patient interviews, structured questionnaires, and review of medical records. Sociodemographic information and patient-reported outcomes were obtained through face-to-face interviews conducted at predefined time points, while clinical and treatment-related data were extracted from hospital electronic medical records. This multimodal data collection approach was used to ensure comprehensive and accurate assessment of clinical characteristics, treatment exposure, and patient-reported outcomes throughout the study period.

The following sociodemographic and clinical variables were systematically collected for all patients: gender (female or male), age category (< 65 years or ≥ 65 years), educational level (primary school, high school, university), economic status (low, moderate, high), social activity status (active or non-active), living arrangement (living alone or living with others), smoking habits (present or absent), alcohol consumption (present or absent), occupational status (active or inactive), presence of comorbidities (yes or no), insulin therapy (yes or no), stoma status (present or absent), primary tumor localization (colorectal, gastric, pancreatic, biliary tract, or esophageal), treatment period (3 months or 6 months), and the occurrence of emergency department visits (yes or no). Occupational status and social activity status were considered distinct variables to reflect different dimensions of patients’ daily functioning. Occupational status was defined as engagement in paid employment, whereas social activity status referred to participation in social interactions and activities outside the home, such as family or community involvement. This distinction allowed for a more comprehensive assessment of patients’ functional and psychosocial characteristics, acknowledging that occupational engagement and social participation may influence well-being through different pathways.

In addition, key clinical characteristics relevant to treatment course and outcomes were systematically recorded, including tumor stage at diagnosis, type of surgical intervention (curative resection with or without stoma formation), and the mode of chemotherapy administration (home-based infusion via port catheter or hospital-based peripheral infusion). These variables were collected to account for potential differences in disease burden, treatment complexity, and care delivery that could influence treatment tolerance, symptom burden, and healthcare utilization.

### Sample size calculation

A formal sample size calculation was performed based on a standard effect size assumption. Assuming a two-sided *α* of 0.05, statistical power of 80%, and a standard effect size (Cohen’s *d*) of 0.5 for the primary outcome (STAI score difference between groups), the required sample size was calculated as 64 patients per group. To account for possible dropouts and missing data, all consecutive eligible patients during the study period were included, resulting in 94 in the home-based port infusion group and 96 in the hospital-based infusion group. This enlarged sample size increased the likelihood of detecting clinically meaningful differences. However, as the study was observational, the power calculation was intended primarily for planning purposes.

### Treatment protocol and follow-up

All patients were scheduled to receive 12 cycles of adjuvant mFOLFOX6 chemotherapy, administered biweekly. The mFOLFOX6 regimen consisted of oxaliplatin at a dose of 85 mg/m^2^ intravenously on day 1, leucovorin 400 mg/m^2^ intravenously on day 1, followed by 5-fluorouracil (5-FU) 400 mg/m^2^ administered as an intravenous bolus, and 2400 mg/m^2^as a continuous infusion over 46 h, repeated every 14 days [21]. The planned duration of adjuvant chemotherapy (3 or 6 months) was determined according to established clinical guidelines and individual patient characteristics, including tumor stage, pathological risk factors, and overall clinical status. Accordingly, a planned treatment duration of 3 months corresponded to six cycles of mFOLFOX6, whereas a 6-month duration consisted of 12 cycles. Patients with lower-risk disease profiles generally received shorter treatment courses, whereas extended treatment duration was favored for those with higher-risk features, in line with contemporary oncologic practice. Treatment duration decisions were made prior to therapy initiation and were not modified based on interim study outcomes. However, shorter treatment duration reflected clinical decisions related to tolerability, toxicity, or patient preference rather than predetermined treatment allocation.

In both treatment groups, chemotherapy was initiated in the hospital on day 1. The distinction between groups was limited to the setting of the subsequent 46-h continuous infusion, which was administered either at home via an ambulatory infusion pump through a port catheter or within the hospital setting using peripheral venous access.

Anxiety and quality-of-life assessments were conducted at three defined time points: at baseline after treatment decision-making but prior to chemotherapy initiation, after the completion of six cycles (approximately 3 months), and again after the completion of 12 cycles (approximately 6 months). Because baseline assessments were performed after treatment decision-making, including port catheter placement, measured anxiety levels may partly reflect patients’ psychological responses to their selected treatment modality. For patients who completed all planned therapy, a final assessment was performed 1 month after treatment conclusion during routine follow-up. Throughout the treatment period, detailed data were recorded at each chemotherapy cycle, including treatment-related toxicities, patient-reported symptoms, and emergency department visits. After six cycles, radiologic staging was performed, and patients without evidence of recurrence or metastasis continued the remaining six cycles as planned. Patients proceeding with therapy were informed of the treatment plan and reassessed for anxiety and quality of life. Following completion of chemotherapy, patients underwent radiologic imaging 1 month later. Patients without recurrence or metastasis were subsequently placed on a routine surveillance schedule with clinic visits every 3 months. Patients with implanted port catheters were advised to attend hospital visits every 3 months for port maintenance and received education on proper port care.

Patient enrollment continued until the predefined sample size was reached. Patients who discontinued treatment prior to completion of baseline assessments were replaced to maintain the target cohort size. Patients who discontinued treatment after baseline assessment due to toxicity, disease progression, comorbidity, or patient preference were not replaced and were excluded from longitudinal and regression analyses.

Emergency department visits were classified based on a review of electronic medical records. An emergency visit was categorized as “without organic pathology” when no objective clinical, laboratory, or radiological findings explaining the presenting symptoms were identified following standard emergency department evaluation. This included cases in which physical examination, routine laboratory tests, and imaging studies, when performed, did not reveal an acute medical condition requiring specific intervention. Classification was based on documented emergency physician assessments and discharge diagnoses recorded in the medical records. No independent adjudication committee was used, and classification relied on routine clinical documentation.

### Measurement tools

Baseline anxiety assessments were conducted after treatment planning decisions, including port catheter placement, but prior to the initiation of chemotherapy. Anxiety levels were assessed using the State–Trait Anxiety Inventory (STAI), originally developed by Spielberger et al. [21]to assess transient and enduring components of anxiety in adult populations. The instrument comprises two subscales: the State Anxiety Scale (STAI-S), measuring situational anxiety, and the Trait Anxiety Scale (STAI-T), assessing general anxiety disposition. Both subscales consist of 20 items scored on a 4-point Likert scale, generating total scores ranging from 20 to 80, where higher scores reflect greater anxiety. Turkish versions of the STAI have been validated and have demonstrated strong reliability in oncology populations [22].

Differences in anxiety levels between patient groups were evaluated based on total STAI-S and STAI-T scores obtained at predefined assessment points. The use of STAI in this context was informed by previous studies comparing psychological outcomes between home-based and hospital-based chemotherapy settings, in which changes in state and trait anxiety were shown to reflect variations in treatment environment, perceived safety, and patient autonomy [21, 22]. Accordingly, STAI scores were analyzed as continuous variables to assess the association of treatment setting with situational and baseline anxiety levels.

Health-related quality of life was evaluated using the European Organization for Research and Treatment of Cancer Quality of Life Questionnaire (EORTC QLQ-C30), originally developed by the EORTC Quality of Life Group [23]for use in cancer populations. This instrument includes functional domains (physical, role, emotional, cognitive, and social functioning), symptom scales (including pain, fatigue, dyspnea, and others), and a global health status scale. Scores range from 0 to 100, with higher scores indicating better functioning for functional domains and greater symptom severity for symptom scales. The Turkish version of the EORTC QLQ-C30 has been validated and is widely used in studies involving patients with cancer [24].

### Statistical analysis

Statistical analyses were performed using IBM SPSS Statistics for Windows, Version 25.0 (IBM Corp., Armonk, NY, USA; licensed software).

Continuous variables were summarized as means and standard deviations, whereas categorical variables were expressed as frequencies and percentages. Intergroup comparisons were conducted using the Mann–Whitney *U* test for non-normally distributed continuous variables and the chi-square test or Fisher’s exact test for categorical variables, as appropriate.

Changes over time within the same group were analyzed using the Friedman test, and post hoc pairwise comparisons were performed with the Wilcoxon signed-rank test when statistically significant differences were identified. These analyses were restricted to patients with complete longitudinal data across all predefined assessment time points. Patients who discontinued treatment due to toxicity, disease progression, or other clinical reasons were not included in longitudinal comparisons, and no imputation procedures were applied. Given the non-normal distribution of the data, non-parametric methods were consistently used for longitudinal analyses. Formal group-by-time interaction effects were not evaluated using parametric repeated-measures models. Bonferroni correction was applied to within-group longitudinal comparisons across multiple time points and to selected between-group comparisons where multiple testing was performed. The use of complete-case analysis may introduce bias, as patients who discontinued treatment were excluded, potentially leading to an underestimation of adverse outcomes.

For regression analyses, emergency department utilization was analyzed as a binary outcome (presence vs. absence of at least one visit during the study period).

Univariable logistic regression analyses were first performed to identify factors associated with these outcomes, and variables with a *p*-value < 0.20 were subsequently entered into multivariable logistic regression models. For logistic regression analyses, dichotomous outcome variables were defined a priori based on clinically meaningful thresholds. Dichotomization of anxiety and quality-of-life measures was performed to enhance clinical interpretability in the regression analyses. High anxiety was defined as a STAI score ≥ 40, in line with commonly used thresholds in psycho-oncology research and to enhance clinical interpretability. Low quality of life was defined as a global health status score below the median value of the study population. Although dichotomization may result in some loss of information, this approach was chosen to facilitate clinically meaningful interpretation of factors associated with adverse psychological and quality-of-life outcomes. Treatment duration was categorized as short term (3 months) or long term (6 months) based on planned chemotherapy duration in accordance with standard adjuvant treatment protocols. Only patients with complete data across all relevant time points were included in regression analyses; no imputation was performed for missing data, as patients with incomplete follow-up were excluded from the analytic cohort. All regression models were therefore based on complete-case analyses. Multicollinearity among variables included in multivariable regression models was assessed using variance inflation factors (VIFs), and no significant multicollinearity was detected. Given the number of variables included relative to the sample size, there is a potential risk of model overfitting.

Odds ratios (ORs) with 95% confidence intervals (CIs) were calculated for all models. Model calibration was assessed using the Hosmer–Lemeshow goodness-of-fit test.

All statistical analyses were conducted on complete-case data, as only patients who completed the planned treatment and assessment schedule were included in the final analysis. No imputation procedures were applied, as missing data were attributable to early treatment discontinuation and not to intermittent missing measurements.

## Results

### Patient characteristics

A total of 190 patients were included in the study, comprising 94 patients who received home-based port infusion therapy and 96 patients who underwent hospital-based infusion. The baseline demographic and clinical characteristics were generally similar between the two groups. There were no significant differences with respect to gender distribution, educational level, economic status, social activity, smoking habits, alcohol consumption, presence of comorbidities, insulin therapy, stoma presence, or primary tumor localization (Supplementary Table 1). Although patients in the home-based port infusion group were initially younger compared to those in the hospital-based infusion group (*p* = 0.014), this difference did not remain statistically significant after Bonferroni correction (adjusted *p* = 0.182). The loss of statistical significance after adjustment suggests that age alone was not significantly associated with the psychosocial outcomes observed in this study. Similarly, living arrangement showed a significant difference in unadjusted analysis (*p* = 0.037), but the significance was lost after adjustment for multiple comparisons (adjusted *p* = 0.481).

**Fig. 1:**
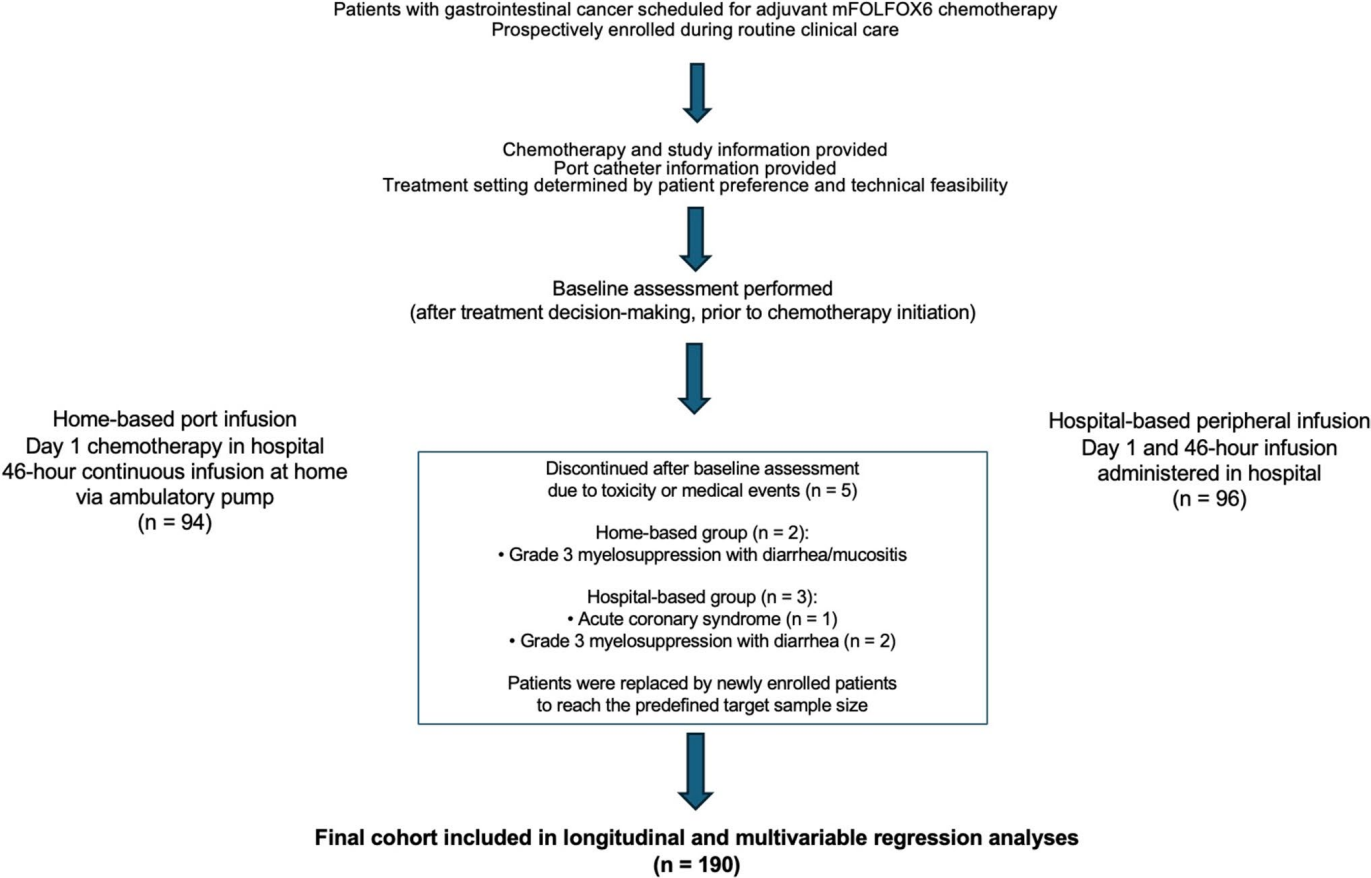
Flow diagram of patient enrollment, treatment allocation, follow-up, and inclusion in the final analysis (Patients were enrolled consecutively as they initiated adjuvant mFOLFOX6 chemotherapy during routine clinical practice. Treatment setting was determined based on patient preference and feasibility of port catheter placement. Patients who discontinued treatment after baseline assessment due to toxicity or medical events were replaced by newly enrolled patients to achieve the predefined target sample size. All analyses were performed on the final cohort with complete baseline assessment)

A detailed flow diagram illustrating patient enrollment, exclusions, discontinuations, and inclusion in the final analyses is provided in Fig. 1.

### Treatment-related adverse events and emergency visits

The incidence of treatment-related adverse events, including nausea, vomiting, diarrhea, constipation, oral mucositis, fatigue, chronic neuropathy, weight loss, neutropenia, anemia, and thrombocytopenia, did not differ significantly between the two treatment groups (Supplementary Table 2). However, emergency department visits for symptoms without an identified organic pathology were reported significantly more frequently in the home-based port infusion group (32%) compared to the hospital-based infusion group (13%) (*p* = 0.002). This difference did not remain statistically significant after Bonferroni correction (adjusted *p* = 0.024) and should therefore be interpreted with caution.

### Changes in anxiety and quality of life over time

In the home-based port infusion group, both state anxiety (STAI-1) and trait anxiety (STAI-2) scores increased significantly following six cycles of chemotherapy compared to baseline (*p* < 0.001 for both), but subsequently decreased to levels below baseline values after 12 cycles (*p* < 0.001 for both) (Supplementary Table 3). Regarding quality of life assessed via the EORTC QLQ-C30, significant declines were observed after six cycles in physical functioning (*p* < 0.001), cognitive functioning (*p* < 0.001), emotional functioning (*p* < 0.001), and global health status (*p* < 0.001). Most of these measures returned to baseline values after 12 cycles, except for minor residual differences that were not statistically significant. Among the symptom scales, dyspnea, pain, fatigue, and nausea/vomiting worsened significantly following six cycles (*p* < 0.001 for all) but showed improvement by the end of 12 cycles.

Conversely, in the hospital-based infusion group, both STAI-1 and STAI-2 scores decreased progressively over time, with significant reductions observed after six and 12 cycles compared to baseline (*p* < 0.001 for all comparisons) (Supplementary Table 4). No significant changes were noted across most functional or symptom scales over time, except for a temporary decline in global health status following six cycles (*p* < 0.001), which subsequently returned to baseline levels after 12 cycles. Among the symptom scales, only diarrhea demonstrated a significant increase after six cycles (*p* < 0.001) in the hospital-based group, with resolution by 12 cycles.

Between-group analyses demonstrated that, after six cycles of chemotherapy, patients receiving home-based port infusion exhibited significantly higher mean scores for both state and trait anxiety compared to those in the hospital-based group (*p* < 0.001 for both), reflecting higher anxiety levels in the home-treated cohort (Table 1). Although differences persisted after 12 cycles, the magnitude of these disparities was attenuated. Additionally, following six cycles, the home-based port infusion group reported significantly higher mean scores for dyspnea (*p* < 0.001), pain (*p* < 0.001), fatigue (*p* = 0.033), and nausea/vomiting (*p* < 0.001) compared to the hospital-based group. Most of these differences diminished by the end of 12 cycles. There were no significant between-group differences observed at any time point regarding role functioning, social functioning, insomnia, or financial difficulties.

**Table 1:** Mean scores of anxiety and quality-of-life measures in patients receiving home-based port infusion versus hospitalized infusion at baseline and after treatment cycles

Variables	Home-based port infusion (*n* = 94)	Hospitalized infusion (*n* = 96)	*p*-value^α^
STAI-1 Baseline After 6 cycles mFOLFOX6 After 12 cycles mFOLFOX6	46.4 ± 26.9 54.4 ± 22.9 39.6 ± 20.2	41.4 ± 24.1 30.2 ± 21.7 26.8 ± 19.6	0.417* **< 0.001** **< 0.001**
STAI-2 Baseline After 6 cycles mFOLFOX6 After 12 cycles mFOLFOX6	46.8 ± 28.6 55.6 ± 24.4 37.9 ± 21.1	46.9 ± 28.6 42.7 ± 22.6 33.4 ± 18.6	0.814 **< 0.001** 0.012*
EORTC QLQ-C30 Functional Scale			
Physical Function Score Baseline After 6 cycles mFOLFOX6 After 12 cycles mFOLFOX6	76.2 ± 14.9 66.7 ± 16.7 76.1 ± 24.1	74.5 ± 15.9 70.2 ± 17.2 76.4 ± 24.6	0.574 0.396* 0.612
Role Function Score Baseline After 6 cycles mFOLFOX6 After 12 cycles mFOLFOX6	78.2 ± 24.6 77.1 ± 24.3 77.2 ± 25.2	79.2 ± 22.3 78.8 ± 26.4 77.9 ± 24.9	0.794 0.812 0.893
Cognitive Function Score Baseline After 6 cycles mFOLFOX6 After 12 cycles mFOLFOX6	80.4 ± 21.5 71.1 ± 22.7 76.4 ± 20.2	80.2 ± 21.8 78.0 ± 25.1 79.4 ± 22.1	0.941 0.326* 0.412*
Emotional Function Score Baseline After 6 cycles mFOLFOX6 After 12 cycles mFOLFOX6	72.2 ± 26.9 60.8 ± 24.6 74.2 ± 24.7	71.9 ± 26.6 67.8 ± 25.4 73.7 ± 25.8	0.745 0.534* 0.813
Social Function Score Baseline After 6 cycles mFOLFOX6 After 12 cycles mFOLFOX6	76.1 ± 20.5 71.9 ± 25.2 74.9 ± 22.1	72.9 ± 22.4 68.8 ± 22.7 73.4 ± 22.6	0.712* 0.609* 0.469
Global Health Status Score Baseline After 6 cycles mFOLFOX6 After 12 cycles mFOLFOX6	62.9 ± 22.3 50.5 ± 30.1 61.9 ± 19.4	62.5 ± 19.4 50.9 ± 30.4 62.4 ± 24.1	0.674 0.712 0.748
EORTC QLQ-C30 Symptom Scales			
Dyspnea Baseline After 6 cycles mFOLFOX6 After 12 cycles mFOLFOX6	9.8 ± 16.7 15.6 ± 25.9 9.7 ± 16.1	9.6 ± 17.4 10.2 ± 17.1 9.8 ± 15.8	0.744 **< 0.001** 0.815
Pain Baseline After 6 cycles mFOLFOX6 After 12 cycles mFOLFOX6	18.0 ± 19.3 30.1 ± 24.9 20.4 ± 21.6	18.1 ± 19.4 20.9 ± 21.1 18.6 ± 19.2	0.869 **< 0.001** 0.464
Fatigue Baseline After 6 cycles mFOLFOX6 After 12 cycles mFOLFOX6	37.9 ± 24.2 49.1 ± 25.4 38.7 ± 24.9	37.6 ± 25.4 42.4 ± 24.5 36.9 ± 24.8	0.769 **0.033** 0.679
Insomnia Baseline After 6 cycles mFOLFOX6 After 12 cycles mFOLFOX6	29.4 ± 31.0 32.2 ± 35.5 31.3 ± 33.3	29.4 ± 31.0 32.2 ± 35.5 30.3 ± 32.7	0.794 0.814 0.849
Loss of appetite Baseline After 6 cycles mFOLFOX6 After 12 cycles mFOLFOX6	16.1 ± 21.4 18.9 ± 29.7 16.4 ± 22.2	16.4 ± 21.2 18.6 ± 29.6 16.9 ± 22.1	0.789 0.812 0.679
Nausea/vomiting Baseline After 6 cycles mFOLFOX6 After 12 cycles mFOLFOX6	13.4 ± 21.9 22.7 ± 25.6 13.1 ± 21.2	13.1 ± 20.4 15.9 ± 18.9 13.2 ± 20.6	0.756 **< 0.001** 0.671
Constipation Baseline After 6 cycles mFOLFOX6 After 12 cycles mFOLFOX6	15.4 ± 26.8 19.8 ± 26.7 17.7 ± 28.2	15.5 ± 27.2 18.8 ± 28.1 17.6 ± 28.4	0.814 0.569 0.796
Diarrhea Baseline After 6 cycles mFOLFOX6 After 12 cycles mFOLFOX6	5.9 ± 14.1 12.7 ± 16.4 6.9 ± 16.1	5.4 ± 14.6 12.5 ± 16.9 6.7 ± 16.4	0.694 0.812 0.759
Financial difficulties Baseline After 6 cycles mFOLFOX6 After 12 cycles mFOLFOX6	25.1 ± 26.7 30.4 ± 26.6 26.4 ± 27.4	26.6 ± 27.9 30.1 ± 26.3 26.8 ± 27.1	0.356 0.534 0.637

Organisation for Research and Treatment of Cancer Quality of Life Questionnaire–Core 30

Between-group comparisons were performed using the Mann–Whitney *U* test. Within-group comparisons were performed using the Friedman test and Wilcoxon signed-rank test. Interaction *p*-values were calculated by repeated-measures ANOVA

* indicates significant difference compared to baseline within group after Bonferroni correction

Values written in bold indicate statistically significant values

### Factors associated with high state anxiety (STAI-1)

In univariable logistic regression analyses, several variables were significantly associated with high levels of state anxiety, including social inactivity (OR, 2.654; 95% CI, 1.814–6.694; *p* = 0.011), living alone (OR, 2.243; 95% CI, 1.637–5.843; *p* = 0.003), home-based port infusion (OR, 2.561; 95% CI, 1.794–4.812; *p* < 0.001), shorter treatment duration of 3 months (OR, 2.613; 95% CI, 1.745–7.156; *p* = 0.033), emergency department visits (OR, 2.348; 95% CI, 1.494–8.346; *p* = 0.044), and low quality of life (OR, 1.948; 95% CI, 1.312–5.456; *p* = 0.034) (Table 2). In the multivariable logistic regression model, social inactivity (OR, 2.749; 95% CI, 1.745–5.337; *p* = 0.003), living alone (OR, 2.494; 95% CI, 1.615–4.619; *p* = 0.002), home-based port infusion (OR, 2.374; 95% CI, 1.796–3.948; *p* < 0.001), shorter treatment duration (OR, 2.418; 95% CI, 1.696–5.948; *p* = 0.022), emergency department visits (OR, 2.456; 95% CI, 1.512–7.946; *p* = 0.037), and low quality of life (OR, 1.933; 95% CI, 1.306–4.967; *p* = 0.034) remained independently associated with high state anxiety, achieving a model fit reflected by a Nagelkerke *R*^2^ value of 0.714.

**Table 2:** Logistic regression analysis for factors associated with high state anxiety (STAI-1)

	Univariable analysis			Multivariable analysis		
	OR	95% CI (lower–upper)	*p*-value	OR	95% CI (lower–upper)	*p*-value
Gender (female)	2.412	0.845–9.612	0.514	-	-	-
Age classification (< 65 year)	2.454	0.769–5.376	0.496	-	-	-
Smoking habits (present)	1.964	0.648–3.961	0.346	-	-	-
Alcohol consumption (present)	1.748	0.745–5.745	0.467	-	-	-
Occupational status (active)	2.156	0.537–7.448	0.512	-	-	-
Social status (non-active)	**2.654**	**1.814–6.694**	**0.011**	**2.749**	**1.745–5.337**	**0.003**
Living alone (single)	**2.243**	**1.637–5.843**	**0.003**	**2.494**	**1.615–4.619**	**0.002**
Type of infusion (home-based port)	**2.561**	**1.794–4.812**	**< 0.001**	**2.374**	**1.796–3.948**	**< 0.001**
Stoma status (present)	2.116	0.764–6.112	0.337	-	-	-
Period (3 months)	**2.613**	**1.745–7.156**	**0.033**	**2.418**	**1.696–5.948**	**0.022**
Emergency department visit (yes)	**2.348**	**1.494–8.346**	**0.044**	**2.456**	**1.512–7.946**	**0.037**
Quality of life (low)	**1.948**	**1.312–5.456**	**0.034**	**1.933**	**1.306–4.967**	**0.034**

These models included multiple variables. Initially, the probability of high state anxiety was 54.6%, but with the developed model, this rate increased to 89.6% (*p* < 0.001, the Nagelkerke *R*^2^ = 0.714). Values in parentheses indicate reference categories used in regression analyses. Dashes (-) indicate variables that were not retained in the multivariable regression model based on the predefined selection criteria

High anxiety was defined as STAI scores ≥ 40. Low quality of life was defined as a global health status score below the median value

Values written in bold indicate statistically significant values

### Factors associated with high trait anxiety (STAI-2)

Univariable analysis revealed significant associations between high trait anxiety and female gender (OR, 2.694; 95% CI, 1.745–6.142; *p* = 0.014), age below 65 years (OR, 2.374; 95% CI, 1.612–4.112; *p* < 0.001), active occupational status (OR, 2.456; 95% CI, 1.537–7.448; *p* = 0.014), social inactivity (OR, 2.654; 95% CI, 1.814–8.694; *p* = 0.011), living alone (OR, 2.348; 95% CI, 1.737–6.948; *p* = 0.003), home-based port infusion (OR, 2.966; 95% CI, 1.814–4.678; *p* < 0.001), presence of a stoma (OR, 2.674; 95% CI, 1.564–9.456; *p* = 0.037), shorter treatment duration (OR, 2.694; 95% CI, 1.745–7.456; *p* = 0.038), emergency department visits (OR, 2.453; 95% CI, 1.569–8.567; *p* = 0.039), and low quality of life (OR, 1.974; 95% CI, 1.296–5.356; *p* = 0.031) (Table 3). All these factors remained significant in the multivariable model, with comparable effect sizes, and the final model exhibited a Nagelkerke *R*^2^ value of 0.708.

**Table 3:** Logistic regression analysis for factors associated with high trait anxiety (STAI-2)

	Univariable analysis			Multivariable analysis		
	OR	95% CI (lower–upper)	*p*-value	OR	95% CI (lower–upper)	*p*-value
Gender (female)	**2.694**	**1.745–6.142**	**0.014**	**2.374**	**1.714–5.679**	**0.021**
Age classification (< 65 year)	**2.374**	**1.612–4.112**	**< 0.001**	**2.281**	**1.512–4.966**	**< 0.001**
Smoking habits (present)	1.796	0.796–4.245	0.641	-	-	-
Alcohol consumption (present)	1.945	0.696–6.747	0.594	-	-	-
Occupational status (active)	**2.456**	**1.537–7.448**	**0.014**	**2.679**	**1.619–5.994**	**0.011**
Social status (non-active)	**2.654**	**1.814–8.694**	**0.011**	**2.966**	**1.745–6.944**	**0.004**
Living alone (single)	**2.348**	**1.737–6.948**	**0.003**	**2.569**	**1.737–5.944**	**0.002**
Type of infusion (home-based port)	**2.966**	**1.814–4.678**	**< 0.001**	**2.948**	**1.752–3.978**	**< 0.001**
Stoma status (present)	**2.674**	**1.564–9.456**	**0.037**	**2.543**	**1.794–8.745**	**0.033**
Period (3 months)	**2.694**	**1.745–7.456**	**0.038**	**2.615**	**1.748–6.964**	**0.031**
Emergency department visit (yes)	**2.453**	**1.569–8.567**	**0.039**	**2.337**	**1.679–7.564**	**0.037**
Quality of life (low)	**1.974**	**1.296–5.356**	**0.031**	**1.871**	**1.337–4.696**	**0.022**

These models included multiple variables. Initially, the probability of high trait anxiety was 54.1%, but with the developed model, this rate increased to 89.9% (*p* < 0.001, the Nagelkerke *R*^2^ = 0.708). Values in parentheses indicate reference categories used in regression analyses. Dashes (-) indicate variables that were not retained in the multivariable regression model based on the predefined selection criteria

High anxiety was defined as STAI scores ≥ 40. Low quality of life was defined as a global health status score below the median value

Values written in bold indicate statistically significant values

### Factors associated with low quality of life

Low quality of life was associated in univariable analyses with social inactivity (OR, 2.694; 95% CI, 1.769–5.948; *p* < 0.001), living alone (OR, 2.319; 95% CI, 1.637–4.948; *p* < 0.001), presence of a stoma (OR, 2.612; 95% CI, 1.748–7.151; *p* = 0.034), shorter treatment duration (OR, 2.494; 95% CI, 1.596–8.204; *p* = 0.041), high state anxiety (OR, 2.678; 95% CI, 1.596–5.678; *p* = 0.021), high trait anxiety (OR, 2.879; 95% CI, 1.811–5.245; *p* = 0.014), and emergency department visits (OR, 1.986; 95% CI, 1.296–4.678; *p* = 0.019) (Table 4). Multivariable analysis confirmed significant associations for social inactivity, living alone, stoma presence, shorter treatment duration, high anxiety scores, and emergency department visits, with the model demonstrating a Nagelkerke *R*^2^ of 0.696.

**Table 4:** Logistic regression analysis for factors associated with low quality of life

	Univariable analysis			Multivariable analysis		
	OR	95% CI (lower–upper)	*p*-value	OR	95% CI (lower–upper)	*p*-value
Gender (female)	2.345	0.741–7.231	0.379	-	-	-
Age classification (< 65 year)	2.114	0.511–5.433	0.456	-	-	-
Smoking habits (present)	**1.948**	**1.796–6.245**	**0.041**	1.748	0.489–7.145	0.496
Alcohol consumption (present)	1.567	0.694–6.112	0.514	-	-	-
Occupational status (active)	**2.252**	**1.437–6.437**	**0.011**	2.145	0.789–5.978	0.267
Social status (non-active)	**2.694**	**1.769–5.948**	**< 0.001**	**2.431**	**1.679–4.564**	**< 0.001**
Living alone (single)	**2.319**	**1.637–4.948**	**< 0.001**	**2.694**	**1.748–4.117**	**< 0.001**
Type of infusion (home-based port)	2.914	0.814–6.174	0.494	-	-	-
Stoma status (present)	**2.612**	**1.748–7.151**	**0.034**	**2.748**	**1.812–6.456**	**0.026**
Period (3 months)	**2.494**	**1.596–8.204**	**0.041**	**2.612**	**1.674–6.945**	**0.031**
STAI-1 (high)	**2.678**	**1.596–5.678**	**0.021**	**2.943**	**1.713–4.966**	**0.022**
STAI-2 (high)	**2.879**	**1.811–5.245**	**0.014**	**2.645**	**1.796–4.569**	**0.019**
Emergency department visit (yes)	**1.986**	**1.296–4.678**	**0.019**	**1.937**	**1.302–4.374**	**0.013**

These models included multiple variables. Initially, the probability of low quality of life was 51.9%, but with the developed model, this rate increased to 84.9% (*p* < 0.001, the Nagelkerke *R*^2^ = 0.696). Values in parentheses indicate reference categories used in regression analyses. Dashes (-) indicate variables that were not retained in the multivariable regression model based on the predefined selection criteria

High anxiety was defined as STAI scores ≥ 40. Low quality of life was defined as a global health status score below the median value

Values written in bold indicate statistically significant values

### Factors associated with emergency department visits without organic pathology

Factors associated with emergency department visits in the absence of identifiable organic pathology included smoking (OR, 2.148; 95% CI, 1.496–4.212; *p* = 0.014), social inactivity (OR, 2.214; 95% CI, 1.569–4.913; *p* < 0.001), living alone (OR, 2.648; 95% CI, 1.712–4.874; *p* < 0.001), home-based port infusion (OR, 2.694; 95% CI, 1.614–4.374; *p* < 0.001), shorter treatment duration (OR, 2.374; 95% CI, 1.633–6.174; *p* < 0.001), high state anxiety (OR, 2.769; 95% CI, 1.744–4.796; *p* < 0.001), high trait anxiety (OR, 2.679; 95% CI, 1.874–4.316; *p* < 0.001), and low quality of life (OR, 2.456; 95% CI, 1.745–8.114; *p* = 0.037) (Table 5). These associations persisted in the multivariable model, which achieved a Nagelkerke *R*^2^ of 0.694, indicating acceptable model fit.

**Table 5:** Logistic regression analysis for factors associated with emergency department visits without an organic pathology diagnosis

	Univariable analysis			Multivariable analysis		
	OR	95% CI (lower–upper)	*p*-value	OR	95% CI (lower–upper)	*p*-value
Gender (female)	1.645	0.641–6.234	0.471	-	-	-
Age classification (< 65 year)	2.414	0.711–6.415	0.404	-	-	-
Smoking habits (present)	**2.148**	**1.496–4.212**	**0.014**	**2.396**	**1.681–4.976**	**0.011**
Alcohol consumption (present)	1.789	0.794–7.645	0.611	-	-	-
Occupational status (active)	1.652	0.537–4.937	0.496	-	-	-
Social status (non-active)	**2.214**	**1.569–4.913**	**< 0.001**	**2.641**	**1.619–4.511**	**< 0.001**
Living alone (single)	**2.648**	**1.712–4.874**	**< 0.001**	**2.614**	**1.698–4.112**	**< 0.001**
Type of infusion (home-based port)	**2.694**	**1.614–4.374**	**< 0.001**	**2.748**	**1.714–4.116**	**< 0.001**
Stoma status (present)	1.948	0.812–7.445	0.537	-	-	-
Period (3 months)	**2.374**	**1.633–6.174**	**< 0.001**	**2.548**	**1.694–5.945**	**< 0.001**
STAI-1 (high)	**2.769**	**1.744–4.796**	**< 0.001**	**2.944**	**1.894–4.116**	**< 0.001**
STAI-2 (high)	**2.679**	**1.874–4.316**	**< 0.001**	**2.637**	**1.745–4.374**	**< 0.001**
Quality of life (low)	**2.456**	**1.745–8.114**	**0.037**	**2.543**	**1.814–9.116**	**0.041**

These models included multiple variables. Initially, the probability of emergency department visits frequency was 54.2%, but with the developed model, this rate increased to 82.7% (*p* < 0.001, the Nagelkerke *R*^2^ = 0.694). Values in parentheses indicate reference categories used in regression analyses. Dashes (-) indicate variables that were not retained in the multivariable regression model based on the predefined selection criteria

High anxiety was defined as STAI scores ≥ 40. Low quality of life was defined as a global health status score below the median value

Values written in bold indicate statistically significant values

## Discussion

This study aimed to compare anxiety levels, quality of life, treatment-related toxicities, and emergency department visits between patients receiving home-based port infusion therapy and those receiving hospital-based infusion. To our knowledge, this is the first prospective study specifically evaluating the psychological and clinical outcomes of home-based port infusion for adjuvant mFOLFOX6 therapy in this patient population, integrating assessments of anxiety, quality of life, and healthcare utilization. Given the observational and non-randomized design of the study, the findings are intended to reflect associations rather than causal relationships between treatment setting and outcomes. These findings provide new insights into the limited literature on psychosocial outcomes associated with home-based chemotherapy for GI malignancies.

Contrary to previous assumptions that patients choosing home-based chemotherapy are likely to be more socially active, independent, and less anxious [25, 26], our results revealed an unexpected pattern. Patients undergoing home-based port infusion experienced significantly higher levels of both state and trait anxiety during the initial treatment phase, despite receiving thorough education and training beforehand. This early rise in anxiety may reflect feelings of uncertainty, social isolation, and the psychological burden of managing prolonged chemotherapy infusions at home without immediate access to healthcare professionals [25–27]. This observed increase in anxiety may reflect contextual factors related to the treatment setting, such as reduced direct clinical supervision and greater responsibility for self-management, rather than representing an intrinsic effect of home-based therapy itself. In the absence of continuous on-site clinical supervision, patients may experience heightened concern regarding symptom interpretation, device-related issues, or the appropriateness of self-management decisions, which may contribute to increased anxiety despite prior education and training [26, 27]. In contrast, the hospital-based group benefited from continuous monitoring and direct interaction with medical staff. Interestingly, anxiety levels among patients treated at home decreased by the end of 12 cycles, suggesting adaptation over time. This temporal pattern, characterized by an initial increase in anxiety followed by subsequent improvement, may reflect a process of psychological adaptation, whereby patients gradually develop familiarity, confidence in self-management, and reduced uncertainty as treatment progresses. Similar concerns have been reported in prior studies indicating that although home-based treatment offers logistical convenience, it may impose greater psychological demands, especially with complex regimens like 46-h infusions [28, 29]. These findings may also reflect practical challenges associated with home-based treatment, including concerns related to infusion device management, uncertainty in recognizing treatment-related symptoms, and limited immediate access to healthcare professionals. Such factors may lower patients’ thresholds for seeking urgent medical evaluation, thereby contributing to higher rates of emergency department visits observed in this group.

Our study found no significant differences in baseline demographic or clinical characteristics between the groups, except for a younger average age in the home-based group, which lost statistical significance after Bonferroni adjustment. This finding suggests that age alone was unlikely to be a primary determinant of anxiety levels. However, despite broadly comparable baseline characteristics, the non-randomized and patient-driven treatment allocation introduces a potential risk of selection bias, and residual confounding cannot be excluded. The relatively balanced baseline characteristics and the consistent effect sizes across multivariable models further suggest that major selection bias is unlikely to fully explain the observed associations.

Although it might be hypothesized that patients declining port catheter placement could represent a subgroup with higher baseline anxiety, this study did not directly assess the reasons underlying patients’ treatment preferences or baseline anxiety prior to treatment initiation. Because reasons for declining port placement or baseline anxiety levels were not systematically assessed, this observation should be interpreted cautiously and cannot be assumed to reflect pre-existing psychological differences between groups. Therefore, no causal inferences can be made regarding the relationship between treatment choice and baseline psychological status. The observed higher anxiety levels among patients receiving home-based therapy should be interpreted in the context of treatment-related factors rather than assumed predispositions, highlighting the complex interplay between treatment setting, patient perceptions, and psychological responses [30].

Regarding treatment-related toxicities, our results align with previous studies indicating that home-based chemotherapy is generally safe and comparable to hospital-based administration in terms of adverse events [31, 32]. However, emergency department visits for symptoms without organic pathology were significantly more frequent in the home-based group. This may reflect heightened anxiety, uncertainty, or a lack of confidence in managing side effects outside the hospital environment. While literature on this topic is limited, some studies suggest that psychosocial factors, including anxiety, contribute to increased unplanned healthcare use among patients receiving home chemotherapy [33]. Notably, this difference did not remain statistically significant after Bonferroni correction, which calls for cautious interpretation of its clinical relevance. In addition, as emergency visits were classified based on routine clinical documentation without independent adjudication, the possibility of misclassification cannot be excluded.

The higher frequency of emergency department visits observed among patients receiving home-based infusion may be explained by several contextual factors inherent to this treatment setting. In the absence of continuous on-site medical supervision, patients may experience increased uncertainty when managing treatment-related symptoms, leading to a lower threshold for seeking urgent medical evaluation. Concerns related to infusion pump function, catheter integrity, or difficulty distinguishing expected treatment effects from potentially serious complications may further contribute to unscheduled healthcare utilization [31, 32]. Additionally, limited immediate access to professional reassurance and varying levels of health literacy may amplify anxiety-driven healthcare-seeking behavior. These findings suggest that, beyond clinical factors, psychosocial and organizational aspects of home-based care play a critical role in shaping patterns of emergency service utilization. Reported reasons for emergency department presentation included concerns regarding infusion pump alarms, catheter-related discomfort, uncertainty about symptom severity, and fear of potential complications.

Quality-of-life assessments revealed temporary declines in the home-based group, particularly after six cycles, affecting physical, cognitive, emotional functioning, and global health status. These impairments largely resolved by the end of 12 cycles, suggesting patient adaptation and potential psychological resilience. Symptom burden including dyspnea, pain, fatigue, and nausea/vomiting also worsened temporarily in the home-based group, consistent with higher anxiety levels observed early in treatment. In contrast, the hospital-based group maintained stable quality-of-life scores over time, except for a transient decline in global health status after six cycles. These findings suggest that patients treated at home may be more sensitive both physically and psychologically during chemotherapy, possibly due to isolation and reduced real-time clinical support. Most prior studies on home chemotherapy have focused primarily on patient satisfaction and logistical advantages [34], whereas detailed longitudinal analyses of psychological and quality-of-life outcomes remain scarce.

Our logistic regression analyses further underscored the psychosocial challenges linked to home-based treatment. Independent factors associated with high state anxiety included social inactivity, living alone, home-based port infusion, shorter treatment duration, emergency department visits, and lower quality of life. Similarly, high trait anxiety was independently associated with female gender, younger age, active employment, social inactivity, living alone, home-based therapy, stoma presence, shorter treatment duration, emergency department visits, and lower quality of life. These findings underscore social isolation and living alone as key contributors to psychological distress during chemotherapy, particularly in patients treated at home. The presence of a stoma, which can significantly impact both physical comfort and social functioning, also emerged as a strong contributor to anxiety [35].

Importantly, lower quality of life was strongly linked to elevated anxiety scores, suggesting a close and potentially bidirectional association between anxiety and diminished quality of life. Other factors independently associated with poor quality of life included social inactivity, living alone, stoma presence, shorter treatment duration, and emergency visits, highlighting the complex psychosocial landscape faced by patients undergoing chemotherapy outside traditional hospital settings [36]. However, given our observational design, we cannot establish definitive causal relationships.

Factors contributing to emergency department visits without an identifiable organic cause included smoking, social inactivity, living alone, home-based treatment, shorter treatment duration, elevated anxiety levels, and lower quality of life. In this context, shorter treatment duration reflected predefined treatment plans based on clinical indications rather than premature discontinuation due to toxicity or patient preference. High anxiety and social isolation may have increased patients’ sensitivity to physical symptoms and uncertainty, thereby lowering thresholds for seeking medical attention. These findings suggest that psychosocial factors, in addition to clinical variables, play an important role in shaping healthcare-seeking behavior among patients receiving home-based chemotherapy, underscoring the need for proactive psychosocial support and structured follow-up in this setting [37].

An additional point that warrants consideration is the potential influence of cultural and healthcare system–related factors on patients’ psychological perceptions of treatment settings. In healthcare environments such as Türkiye, where hospital-based supportive care remains relatively accessible and where direct interaction with healthcare professionals is strongly valued by patients, inpatient or hospital-centered treatment may provide a greater sense of safety, emotional reassurance, and immediate professional support. Previous studies have emphasized that patients’ perceptions of safety and confidence during cancer treatment are closely influenced by communication with healthcare providers, accessibility of supportive care, and monitoring practices [29, 31]. Therefore, the higher anxiety levels observed among patients receiving home-based infusion in the early treatment phase should not necessarily be interpreted as evidence that home-based chemotherapy is universally inferior or that transition toward outpatient care should always be promoted regardless of context. Rather, these findings highlight the importance of considering local healthcare infrastructure, patient expectations, family support systems, and cultural perceptions of medical care when implementing home-based oncology services.

Our findings further suggest that the psychological burden associated with home-based chemotherapy may potentially be mitigated through structured supportive interventions, including regular telephone follow-up, rapid-access consultation systems, telemonitoring approaches, and enhanced patient and caregiver education regarding symptom management and device-related concerns. Prior studies have suggested that structured communication and supportive care interventions may improve patients’ sense of security and reduce distress-related healthcare utilization among patients receiving outpatient cancer treatment [31, 33]. Such supportive strategies may improve patients’ sense of safety and confidence during home treatment and could reduce anxiety-driven emergency department utilization. The continuous availability of healthcare professionals in hospital-based settings may reduce uncertainty related to symptom interpretation and device-related concerns, thereby contributing to greater patient reassurance during treatment. Future studies evaluating home-based chemotherapy within integrated supportive care models may help clarify whether psychological outcomes differ once reassurance and accessibility comparable to hospital-based care are established.

The strengths of our study include its prospective design, relatively large sample size, and comprehensive evaluation of psychological and clinical outcomes across multiple time points. However, given the observational nature of the study, causal relationships cannot be inferred. The associations observed should be interpreted as correlational, and residual confounding cannot be excluded despite multivariable adjustment. Despite the strengths of the present study, several limitations should be acknowledged. First, the non-randomized design may have introduced selection bias, as treatment allocation was influenced by patient preference and clinical considerations rather than random assignment. Although baseline characteristics were largely comparable between groups, unmeasured confounders may have influenced both treatment selection and outcomes. Second, the study was conducted at a single center, which may limit the generalizability of the findings to other healthcare settings with different organizational structures or patient populations. Third, baseline anxiety levels were not assessed prior to treatment decision-making, including port catheter placement; therefore, changes in anxiety over time should be interpreted cautiously, as pre-existing psychological vulnerability may have influenced both treatment selection and baseline anxiety levels. In addition, treatment duration differed between patients based on clinical decision-making and treatment tolerance rather than random allocation, and shorter treatment duration may reflect a combination of disease-related factors, treatment-related toxicity, or patient preference rather than a single underlying mechanism. Furthermore, although comprehensive data collection was performed, the observational design precludes causal inference, and residual confounding may persist despite multivariable adjustment. Finally, while the study provides insight into real-world practice, the lack of standardized psychosocial interventions and structured remote support may have influenced patient-reported outcomes. Although anxiety and quality-of-life measures were analyzed as continuous variables in descriptive and longitudinal analyses, dichotomized outcomes were used in regression models to improve clinical interpretability, which may have reduced statistical power. In addition, although linear mixed-effects models could provide a more flexible framework for repeated-measures data, non-parametric methods were preferred due to the non-normal distribution of the variables and the complete-case structure of the dataset. Moreover, the use of complete-case analysis may have introduced bias by excluding patients who discontinued treatment, potentially leading to an underestimation of adverse outcomes. Additionally, given the number of variables included in the regression models relative to the sample size, the possibility of model overfitting should be considered when interpreting the results.

Future multicenter, prospective studies incorporating standardized baseline psychological assessment, structured psychosocial support programs, and telemedicine-based follow-up are warranted to further clarify the relationship between treatment setting, psychological well-being, and clinical outcomes. Although structured psychosocial or telemonitoring interventions were not formally implemented in this study, patients received standard education regarding port care, symptom recognition, and when to seek medical attention. The absence of a structured follow-up or remote monitoring program may have contributed to increased anxiety during the early treatment phase, highlighting the potential value of integrating systematic supportive care models into home-based chemotherapy pathways.

## Conclusion

This study demonstrated that home-based port infusion for adjuvant mFOLFOX6 therapy in patients with gastrointestinal cancers is associated with transient increases in anxiety and temporary declines in certain aspects of quality of life, especially during the early treatment phase. However, these adverse effects tended to resolve as treatment progressed, indicating that patients gradually adapt. These findings highlight the importance of carefully designing home-based chemotherapy programs that address not only medical but also psychosocial needs, while taking into account healthcare systems, cultural expectations, and available supportive care resources. Despite the important logistical advantages of home-based infusion therapy, its psychological impact may vary according to the level of structured support and patients’ perceived access to professional care. Because randomization was not feasible due to ethical considerations, multivariable analyses were performed to minimize potential confounding, and the groups remained largely comparable at baseline. Nevertheless, given the non-randomized design, these findings should be interpreted as associations rather than causal effects. Notably, the psychological burden of home-based therapy appears more pronounced among patients who live alone or are socially isolated, potentially contributing to higher rates of emergency department visits. Therefore, integrating targeted psychosocial support and systematic follow-up into home-based chemotherapy programs for this vulnerable group is essential. Additionally, educational interventions and easily accessible telephone counseling services may help reduce anxiety and prevent unnecessary emergency visits in patients receiving home-based treatment. Our findings shed light on important psychosocial dimensions that must be considered home-based infusion therapy becomes increasingly common, underscoring the need for future larger, randomized, multicenter studies to confirm these results and inform patient-centered care strategies.

## Supplementary Information

Below is the link to the electronic supplementary material.

ESM 1 (DOCX 26.6 KB)

## Data Availability

No datasets were generated or analysed during the current study.

## References

[1] Kulthanachairojana N, Chansriwong P, Thokanit NS, Sirilerttrakul S, Wannakansophon N, Taychakhoonavudh S (2021) Home-based chemotherapy for stage III colon cancer patients in Thailand: cost-utility and budget impact analyses. Cancer Med 10(3):1027–1033. 10.1002/cam4.3690 33377629 10.1002/cam4.3690 PMC7897966

[2] Villegas E, Arruñada M, Casado MÁ, González S, Moreno-Martínez ME et al (2024) National expert consensus on home-administered oncologic therapies in Spain. Front Oncol 14:1335344. 10.3389/fonc.2024.1335344 38434688 10.3389/fonc.2024.1335344 PMC10905380

[3] Kim HS, Kim JH, Kim HJ, Jang HJ, Kim JB et al (2012) Oxaliplatin, 5-fluorouracil and leucovorin (modified FOLFOX-6) as first-line chemotherapy for advanced gastric cancer patients with poor performance status. Oncol Lett 3(2):425–428. 10.3892/ol.2011.496 22740925 10.3892/ol.2011.496 PMC3362383

[4] Folprecht G, Pericay C, Saunders MP, Thomas A, Lopez Lopez R et al (2016) Oxaliplatin and 5-FU/folinic acid (modified FOLFOX6) with or without aflibercept in first-line treatment of patients with metastatic colorectal cancer: the AFFIRM study. Ann Oncol 27(7):1273–1279. 10.1093/annonc/mdw176 27091810 10.1093/annonc/mdw176

[5] Gustavsson B, Carlsson G, Machover D, Petrelli N, Roth A et al (2015) A review of the evolution of systemic chemotherapy in the management of colorectal cancer. Clin Colorectal Cancer 14(1):1–10. 10.1016/j.clcc.2014.11.002 25579803 10.1016/j.clcc.2014.11.002

[6] Vodenkova S, Buchler T, Cervena K, Veskrnova V, Vodicka P, Vymetalkova V (2020) 5-fluorouracil and other fluoropyrimidines in colorectal cancer: past, present and future. Pharmacol Ther 206:107447. 10.1016/j.pharmthera.2019.107447 31756363 10.1016/j.pharmthera.2019.107447

[7] Quesada S, Samalin E, Thezenas S, Khellaf L, Mourregot A et al (2022) Perioperative FOLFOX in patients with locally advanced oesogastric adenocarcinoma. Anticancer Res 42(1):185–193. 10.21873/anticanres.15472 34969724 10.21873/anticanres.15472

[8] Lee J, Hur SM, Kim Z, Lim CW (2021) Safety of immediate use of totally implantable venous access ports in adult patients with cancer: a retrospective single-center study. Korean J Clin Oncol 17(2):104–110. 10.14216/kjco.21016 36945672 10.14216/kjco.21016 PMC9942755

[9] Larsen FO, Christiansen AB, Rishøj A, Nelausen KM, Nielsen DL (2018) Safety and feasibility of home-based chemotherapy. Dan Med J 65(5):A5482 29726319

[10] Erişen MA, Yılmaz FÖ (2023) Comparison of chemotherapy treatment administration via venous port and peripheral vascular access in terms of quality of life and costs. Qual Life Res 32(7):1897–1908. 10.1007/s11136-023-03365-6 36790666 10.1007/s11136-023-03365-6 PMC9930058

[11] Duggan C, Hernon O, Dunne R, McInerney V, Walsh SR et al (2024) Vascular access device type for systemic anti-cancer therapies in cancer patients: a scoping review. Crit Rev Oncol Hematol 196:104277. 10.1016/j.critrevonc.2024.104277 38492760 10.1016/j.critrevonc.2024.104277

[12] Polinski JM, Kowal MK, Gagnon M, Brennan TA, Shrank WH (2017) Home infusion: safe, clinically effective, patient preferred, and cost saving. Healthcare Basel 5(1–2):68–80. 10.1016/j.hjdsi.2016.04.004 28668202 10.1016/j.hjdsi.2016.04.004

[13] Kelly D, Pearce S, Butters E, Stevens W, Layzell S (2004) Achieving change in the NHS: a study to explore the feasibility of a home-based cancer chemotherapy service. Int J Nurs Stud 41(2):215–224. 10.1016/j.ijnurstu.2003.05.002 14725786 10.1016/j.ijnurstu.2003.05.002

[14] Witwaranukool P, Phonyiam R, Wu Y, Kynoch K (2024) Experiences and perceptions of patients with cancer receiving home-based chemotherapy: a qualitative systematic review protocol. Syst Rev 13(1):242. 10.1186/s13643-024-02659-1 39342375 10.1186/s13643-024-02659-1 PMC11438387

[15] Jiang Y, Zhao M, Tang W, Zheng X (2024) Impacts of systemic treatments on health-related quality of life for patients with metastatic colorectal cancer: a systematic review and network meta-analysis. BMC Cancer 24(1):188. 10.1186/s12885-024-11937-z 38336718 10.1186/s12885-024-11937-z PMC10854105

[16] Sirilerttrakul S, Wannakansophon N, Utthiya P, Ckumdee S, Tangteerakoon P, Chansriwong P (2021) Evaluation of adverse events and health-related quality of life in patients with colorectal cancer receiving ambulatory home-based chemotherapy in Thailand. Nurs Open 8(6):3036–3044. 10.1002/nop2.1016 34382364 10.1002/nop2.1016 PMC8510724

[17] Shi L, Zhang J, Deng Y (2025) Associations of pretreatment emotional distress with adherence to therapy for patients with locally advanced rectal cancer: a post hoc analysis of the Chinese FOWARC phase 3 randomized clinical trial. BMC Med 23(1):293. 10.1186/s12916-025-04128-5 40399932 10.1186/s12916-025-04128-5 PMC12096767

[18] Dixit J, Gupta N, Kataki A, Roy P, Mehra N et al (2024) Health-related quality of life and its determinants among cancer patients: evidence from 12,148 patients of Indian database. Health Qual Life Outcomes 22(1):26. 10.1186/s12955-024-02227-0 38481231 10.1186/s12955-024-02227-0 PMC10938809

[19] Shalata W, Gothelf I, Bernstine T, Michlin R, Tourkey L et al (2024) Mental health challenges in cancer patients: a cross-sectional analysis of depression and anxiety. Cancers (Basel) 16(16):2827. 10.3390/cancers16162827 39199598 10.3390/cancers16162827 PMC11352929

[20] Karovic S, Dvergsten E, Pierattini C, Barac A, Fay L et al (2025) Real-world data on risk factors for emergency department visits to treat outpatient chemotherapy-associated toxicities. Cancer Res Commun 5(6):973–980. 10.1158/2767-9764.CRC-24-0631 40464592 10.1158/2767-9764.CRC-24-0631 PMC12169210

[21] Spielberger CD, Gorsuch RL, Lushene R, Vagg PR, Jacobs GA (1983) Manual for the State-Trait Anxiety Inventory. Consulting Psychologists Press, Palo Alto, CA

[22] Oner N, Le Compte A (1985) Durumluk-Surekli kaygı envanteri el kitabı. Boğaziçi Yayınları, Istanbul

[23] Aaronson NK, Ahmadzai S, Bergman B, Bullinger M, Cull A, Duez NJ et al (1993) The European Organization for Research and Treatment of Cancer QLQ-C30: a quality-of-life instrument for use in international clinical trials in oncology. J Natl Cancer Inst 85(5):365–376 8433390 10.1093/jnci/85.5.365

[24] Demirci S, Eser E, Ozsaran Z, Tankisi D, Aras AB et al (2011) Validation of the Turkish versions of EORTC QLQ-C30 and BR23 modules in breast cancer patients. Asian Pac J Cancer Prev 12(5):1283–1287 21875283

[25] Kok A, Passchier E, May AM, Jager-Wittenaar H, Veenhof C et al (2024) Expectations and experiences of participating in a supervised and home-based physical exercise intervention in patients with head and neck cancer during chemoradiotherapy: a qualitative study. Curr Oncol 31(2):885–899 38392060 10.3390/curroncol31020066 PMC10887739

[26] Seiler A, Jenewein J (2019) Resilience in cancer patients. Front Psychiatry 10:208. 10.3389/fpsyt.2019.00208 31024362 10.3389/fpsyt.2019.00208 PMC6460045

[27] Ike KGO, de Boer SF, Buwalda B, Kas MJH (2020) Social withdrawal: an initially adaptive behavior that becomes maladaptive when expressed excessively. Neurosci Biobehav Rev 116:251–267. 10.1016/j.neubiorev.2020.06.030 32610177 10.1016/j.neubiorev.2020.06.030

[28] Ferro S, Serra C (2025) Triage at shift changes and distortions in the perception and treatment of emergency patients. J Health Econ 99:102944. 10.1016/j.jhealeco.2024.102944 39657376 10.1016/j.jhealeco.2024.102944

[29] Areia C, King E, Ede J, Young L, Tarassenko L, Watkinson P, Vollam S (2022) Experiences of current vital signs monitoring practices and views of wearable monitoring: a qualitative study in patients and nurses. J Adv Nurs 78(3):810–822. 10.1111/jan.15055 34655093 10.1111/jan.15055 PMC9293408

[30] Yeo HY, Liew AC, Chan SJ, Anwar M, Han CH, Marra CA (2023) Understanding patient preferences regarding the important determinants of breast cancer treatment: a narrative scoping review. Patient Prefer Adherence 17:2679–2706. 10.2147/PPA.S432821 37927344 10.2147/PPA.S432821 PMC10625390

[31] Stiefel F, Bourquin C, Salmon P, Achtari Jeanneret L, Dauchy S, ESMO Guidelines Committee et al (2024) Communication and support of patients and caregivers in chronic cancer care: ESMO Clinical Practice Guideline. ESMO Open 9(7):103496. 10.1016/j.esmoop.2024.103496 39089769 10.1016/j.esmoop.2024.103496 PMC11360426

[32] Wang TQ, Samuel JN, Brown MC, Vennettilli A, Solomon H et al (2018) Routine surveillance of chemotherapy toxicities in cancer patients using the patient-reported outcomes version of the Common Terminology Criteria for Adverse Events (PRO-CTCAE). Oncol Ther 6(2):189–201. 10.1007/s40487-018-0065-7 32700029 10.1007/s40487-018-0065-7 PMC7360011

[33] Van Beek FE, Wijnhoven LMA, Holtmaat K, Custers JAE, Prins JB et al (2021) Psychological problems among cancer patients in relation to healthcare and societal costs: a systematic review. Psychooncology 30(11):1801–1835. 10.1002/pon.5753 34228838 10.1002/pon.5753 PMC9291760

[34] Corbett M, Heirs M, Rose M, Smith A, Stirk L, Richardson G, Stark D, Swinson D, Craig D, Eastwood A (2015Apr 24) The delivery of chemotherapy at home: an evidence synthesis. Health and Social Care Delivery Research. 3(14):1–82 25927120

[35] Ayaz-Alkaya S (2019) Overview of psychosocial problems in individuals with stoma: a review of literature. Int Wound J 16(1):243–249. 10.1111/iwj.13018 30392194 10.1111/iwj.13018 PMC7948730

[36] Rat LA, Ghitea TC, Maghiar AM (2025) Psychological distress and quality of life in patients with colon cancer: predictors, moderating effects, and longitudinal impact. Healthcare Basel 13(7):753. 10.3390/healthcare13070753 40218051 10.3390/healthcare13070753 PMC11988287

[37] Pollock A, Campbell P, Cheyne J, Cowie J, Davis B et al (2020) Interventions to support the resilience and mental health of frontline health and social care professionals during and after a disease outbreak, epidemic or pandemic: a mixed methods systematic review. Cochrane Database Syst Rev 11(11):CD013779. 10.1002/14651858.CD013779 33150970 10.1002/14651858.CD013779 PMC8226433

