# Supplementary material for: Impact of home-based port infusion versus hospital-based peripheral infusion on anxiety, quality of life, and emergency visits in gastrointestinal cancer: a prospective observational study by the Turkish Oncology Group (TOG)
Source: Support Care Cancer. 2026 May 22;34(6):569. doi: 10.1007/s00520-026-10790-z (PMC13197445; doi:10.1007/s00520-026-10790-z)
Supplement: Supplementary file 1 — (DOCX 26.6 KB) [file 520_2026_10790_MOESM1_ESM.docx]

**Supplementary Table 1. Comparison of baseline demographic and clinical characteristics between patients receiving home-based port infusion and hospitalized infusion.**

| **Variables** | **Home-based port infusion**  **(n=94)** | **Hospitalized infusion**  **(n=96)** | **P**  **value ^α^** | **Bonferroni-adjusted p value ^*^** |
| --- | --- | --- | --- | --- |
| **Gender; n (%)**  Female  Male | 40 (43)  54 (57) | 44 (46)  52 (54) | 0.348 |  |
| **Age classification; n (%)**  <65 year  ≥65 year | 64 (68)  30 (32) | 37 (39)  59 (61) | **0.014** | 0.182 |
| **Living arrangement; n (%)**  Lives alone  Lives with spouse/partner  Lives with children/relatives | 2 (2)  48 (51)  44 (47) | 13 (14)  51 (53)  32 (33) | **0.037** | 0.481 |
| **Occupational status; n (%)**  Unemployed/housewife/retired  Active worker  On medical leave | 12 (13)  37 (39)  45 (48) | 24 (25)  33 (34)  39 (41) | 0.296 |  |
| **Educational status; n (%)**  Low  High | 64 (68)  30 (32) | 59 (61)  37 (39) | 0.414 |  |
| **Economic status; n (%)**  Low/Middle  High | 62 (66)  32 (34) | 58 (60)  38 (40) | 0.496 |  |
| **Social status; n (%)**  Socially active  Socially restricted/isolated | 48 (51)  46 (49) | 46 (48)  50 (52) | 0.474 |  |
| **Smoking habits; n (%)**  Present  Absent | 48 (51)  46 (49) | 37 (39)  59 (61) | 0.116 |  |
| **Alcohol consumption; n (%)**  Present  Absent | 24 (26)  70 (74) | 19 (20)  77 (80) | 0.294 |  |
| **Comorbidity n (%)**  Present  Absent | 33 (35)  61 (65) | 37 (39)  59 (61) | 0.412 |  |
| **Insulin treatment n (%)**  Present  Absent | 11 (12)  83 (88) | 11 (12)  85 (88) | 0.506 |  |
| **Stoma status n (%)**  Present  Absent | 14 (15)  80 (85) | 19 (20)  77 (80) | 0.483 |  |
| **Primary tumor localization; n (%)**  Colon  Rectum  Stomach  Pancreas  Esophagus | 48 (51)  22 (23)  14 (15)  6 (6)  4 (5) | 46 (48)  20 (21)  18 (19)  8 (8)  4 (4) | 0.468 |  |

^α^ Since the data are not normally distributed, the Mann-Whitney U test was used for comparison between groups. Significant p values are shown in bold (p<0.005). However, Bonferroni-corrected significance threshold: p < 0.00385.

**Supplementary Table 2. Comparison of treatment-related adverse events between patients receiving home-based port infusion and hospitalized infusion.**

| **Variables** | **Home-based port infusion**  **(n=94)** | **Hospitalized infusion**  **(n=96)** | **P**  **value ^α^** | **Bonferroni-adjusted p value ^*^** |
| --- | --- | --- | --- | --- |
| **Nausea; n (%)**  Absent  Grade 1-2 | 57 (61)  37 (39) | 63 (66)  33 (34) | 0.596 |  |
| **Vomiting; n (%)**  Absent  Grade 1-2 | 46 (49)  48 (51) | 51 (53)  45 (47) | 0.374 |  |
| **Diarrhea; n (%)**  Absent  Grade 1-2 | 62 (66)  32 (34) | 64 (67)  32 (33) | 0.348 |  |
| **Constipation; n (%)**  Absent  Grade 1-2 | 46 (49)  48 (51) | 51 (53)  45 (47) | 0.374 |  |
| **Oral Mucositis; n (%)**  Absent  Grade 1-2 | 54 (57)  40 (43) | 58 (60)  38 (40) | 0.348 |  |
| **Fatigue; n (%)**  Absent  Grade 1-2 | 46 (49)  48 (51) | 51 (53)  45 (47) | 0.374 |  |
| **Chronic neuropathy; n (%)**  Absent  Grade 1-2 | 57 (61)  37 (39) | 63 (66)  33 (34) | 0.412 |  |
| **Weight loss*; n (%)**  Absent  Present | 64 (68)  30 (32) | 58 (60)  38 (40) | 0.074 |  |
| **Neutropenia; n (%)**  Absent  Grade 1-2 | 57 (61)  37 (39) | 63 (66)  33 (34) | 0.412 |  |
| **Anemia; n (%)**  Absent  Grade 1-2 | 54 (57)  40 (43) | 58 (60)  38 (40) | 0.348 |  |
| **Thrombocytopenia; n (%)**  Absent  Grade 1-2 | 61 (65)  33 (35 | 62 (65)  34 (35) | 0.844 |  |
| **Admission to emergency department (no organic pathology detected)** ; n (%)**  Absent  Present | 64 (68)  30 (32) | 84 (87)  12 (13) | **0.002** | 0.024 |

**Palpitations, chest pain, subjective fever, chills, dyspnea, abdominal pain, dizziness.

α Bonferroni correction applied; adjusted α = 0.00417. * indicates significant difference vs baseline after Bonferroni correction.

**Supplementary Table 3. Changes in anxiety and quality-of-life scores over time in patients receiving home-based port infusion.**

| **Variables** | **Baseline** | **After 6 cycles mFOLFOX6** | **p Value within-group**  **(vs baseline)** | **After 12 cycles mFOLFOX6** | **p Value within-group**  **(vs baseline)** |
| --- | --- | --- | --- | --- | --- |
| **STAI- 1** | 46.4±26.9 | 54.4±22.9 | **<0.001** | 39.6±20.2 | **<0.001** |
| **STAI- 2** | 46.8±28.6 | 55.6±24.4 | **<0.001** | 37.9±21.1 | **<0.001** |
| **EORTC QLQ-C30 Functional Scale** | | | | | |
| Physical Function Score | 76.2±14.9 | 66.7±16.7 | **<0.001** | 76.1±24.1 | 0.435 |
| Role Function Score | 78.2±24.6 | 77.1±24.3 | 0.748 | 77.2±25.2 | 0.814 |
| Cognitive Function Score | 80.4±21.5 | 71.1±22.7 | **<0.001** | 76.4±20.2 | 0.205 |
| Emotional Function Score | 72.2±26.9 | 60.8±24.6 | **<0.001** | 71.2±24.7 | 0.569 |
| Social Function Score | 76.1±20.5 | 71.9±25.2 | 0.148 | 74.9±22.1 | 0.314 |
| Global Health Status Score | 62.9±22.3 | 50.5±30.1 | **<0.001** | 61.9±19.4 | 0.412 |
| **EORTC QLQ-C30 Symptom Scales** | | | | | |
| Dyspnea | 9.8±16.7 | 15.6±25.9 | **<0.001** | 9.7±16.1 | 0.594 |
| Pain | 18.0±19.3 | 30.1±24.9 | **<0.001** | 20.4±21.6 | 0.337 |
| Fatigue | 37.9±24.2 | 49.1±25.4 | **<0.001** | 38.7±24.9 | 0.512 |
| Insomnia | 29.4±31.0 | 32.2±35.5 | 0.137 | 31.3±33.3 | 0.196 |
| Loss of appetite | 16.1±21.4 | 18.9±29.7 | 0.109 | 16.4±22.2 | 0.619 |
| Nausea/Vomiting | 13.4±21.9 | 22.7±25.6 | **<0.001** | 13.1±21.2 | 0.766 |
| Constipation | 15.4±26.8 | 19.8±26.7 | 0.411* | 17.7±28.2 | 0.369 |
| Diarrhea | 5.9±14.1 | 12.7±16.4 | **<0.001** | 6.9±16.1 | 0.297 |
| Financial Difficulties | 25.1±26.7 | 30.4±26.6 | 0.524* | 26.4±27.4 | 0.496 |

Between-group comparisons were performed using the Mann-Whitney U test. Within-group comparisons were performed using the Friedman test and Wilcoxon signed-rank test. **Interaction p-values were calculated by repeated measures ANOVA.** Bonferroni correction applied where appropriate. * indicates significant difference vs baseline after Bonferroni correction.

.

**Supplementary Table 4. Changes in anxiety and quality-of-life scores over time in patients receiving hospitalized infusion.**

| **Variables** | **Baseline** | **After 6 cycles mFOLFOX6** | **p Value within-group**  **(vs baseline)** | **After 12 cycles mFOLFOX6** | **p Value within-group**  **(vs baseline)** |
| --- | --- | --- | --- | --- | --- |
| **STAI- 1** | 41.4±24.1 | 30.2±21.7 | **<0.001** | 26.8±19.6 | **<0.001** |
| **STAI- 2** | 46.9±28.6 | 42.7±22.6 | **<0.001** | 33.4±18.6 | **<0.001** |
| **EORTC QLQ-C30 Functional Scale** | | | | | |
| Physical Function Score | 74.5±15.9 | 70.2±17.2 | 0.196 | 76.4±24.6 | 0.314 |
| Role Function Score | 79.2±22.3 | 78.8±26.4 | 0.814 | 77.9±24.9 | 0.196 |
| Cognitive Function Score | 80.2±21.8 | 78.0±25.1 | 0.116 | 79.4±22.1 | 0.474 |
| Emotional Function Score | 71.9±26.6 | 67.8±25.4 | **0.033*** | 73.7±25.8 | 0.312 |
| Social Function Score | 72.9±22.4 | 68.8±22.7 | 0.069 | 73.4±22.6 | 0.511 |
| Global Health Status Score | 62.5±19.4 | 50.9±30.4 | **<0.001** | 62.4±24.1 | 0.843 |
| **EORTC QLQ-C30 Symptom Scales** | | | | | |
| Dyspnea | 9.6±17.4 | 10.2±17.1 | 0.376 | 9.8±15.8 | 0.462 |
| Pain | 18.1±19.4 | 20.9±21.1 | 0.545 | 18.6±19.2 | 0.583 |
| Fatigue | 37.6±25.4 | 42.4±24.5 | 0.116 | 36.9±24.8 | 0.396 |
| Insomnia | 29.4±31.0 | 32.2±35.5 | 0116 | 30.3±32.7 | 0.512 |
| Loss of appetite | 16.4±21.2 | 18.6±29.6 | 0.122 | 16.9±22.1 | 0.694 |
| Nausea/Vomiting | 13.1±20.4 | 15.9±18.9 | 0.316 | 13.2±20.6 | 0.911 |
| Constipation | 15.5±27.2 | 18.8±28.1 | 0.512* | 17.6±28.4 | 0.433 |
| Diarrhea | 5.4±14.6 | 12.5±16.9 | **<0.001** | 6.7±16.4 | 0.272 |
| Financial Difficulties | 26.6±27.9 | 30.1±26.3 | 0.494* | 26.8±27.1 | 0.315 |

Between-group comparisons were performed using the Mann-Whitney U test. Within-group comparisons were performed using the Friedman test and Wilcoxon signed-rank test. **~~Interaction p-values were calculated by repeated measures ANOVA.~~** Bonferroni correction applied where appropriate. * indicates significant difference vs baseline after Bonferroni correction.
